# The Reason Why

**Published:** 1887-05

**Authors:** 


THE REASON WHY.—It was our intention to issue a daily edition of the JOURNAL during the session of the Association, but seeing, the first day that only 93 answered to roll call, we gave it out; and it is fortunate that we did, for not one moment of time did we have to give to such an enterprise. Having no assistant it was a matter of physical as well as moral impossibility, unless we could have been ubiquitous, and endowed with as many hands as Briarius is said to hav? had arms.

---

The usefulness of good Hypophosphites in Pulmonary and Strumous affections is generally agreed upon by the Profession.

We commend to the notice of our readers the advertisement on page —— of this number. "ROBINSON'S HYPOPHOSPHITES" is an elegant and uniformly active preparation; the presence in it of Quinine, Strychnine, Iron, etc., adding highly to its tonic value.

